# Hechtian Strands Transmit Cell Wall Integrity Signals in Plant Cells

**DOI:** 10.3390/plants9050604

**Published:** 2020-05-09

**Authors:** Arata Yoneda, Misato Ohtani, Daisuke Katagiri, Yoichiroh Hosokawa, Taku Demura

**Affiliations:** 1 Graduate School of Science and Technology, Nara Institute of Science and Technology, Ikoma 630-0192, Japan; arata.yoneda@gmail.com (A.Y.); misato@edu.k.u-tokyo.ac.jp (M.O.); katagiri.daisuke1990@gmail.com (D.K.); hosokawa@ms.naist.jp (Y.H.); 2 Department of Integrated Biosciences, Graduate School of Frontier Sciences, The University of Tokyo, Kashiwa 277-8562, Japan

**Keywords:** Hechtian strands, plasma membrane, cell wall, plasmolysis, femtosecond laser microdissection, cell wall regeneration

## Abstract

Hechtian strands are thread-like structures in plasmolyzed plant cells that connect the cell wall to the plasma membrane. Although these strands were first observed more than 100 years ago, their physiological roles are largely unknown. Here, we used intracellular laser microdissection to examine the effects of disrupting Hechtian strands on plasmolyzed tobacco BY-2 cells. When we focused femtosecond laser pulses on Hechtian strands, targeted disruptions were induced, but no visible changes in cell morphology were detected. However, the calcofluor white signals from β-glucans was detected in plasmolyzed cells with disrupted Hechtian strands, whereas no signals were detected in untreated plasmolyzed cells. These results suggest that Hechtian strands play roles in sensing cell wall integrity.

## 1. Introduction

Under high osmotic conditions, plant cells lose water and shrink, causing the plasma membrane to detach from the cell wall. This process, termed plasmolysis, is a characteristic response of plant cells to osmotic stress. During plasmolysis, the plasma membrane does not separate from the cell wall completely; extended membranous threads called Hechtian strands (described by Hecht in 1912, [1]) firmly connect the plasma membrane to the cell wall [2,3,4,5]. Although Hechtian strands were first observed more than 100 years ago [1], our knowledge of their physical properties is limited to the following findings: 1) Hechtian strands are 30 to 250 nm in diameter [3,6]; 2) the length and density of these strands vary depending on the cell and experimental condition [1,2,3,4,5,6,7,8,9]; and 3) the density, length, and tension of Hechtian strands can be altered by cold hardening treatment [9,10]. Biochemical assays indicated that Hechtian strands contain cytoplasm and/or endoplasmic reticulum (ER) in their interior regions [3,11,12], and the constricted tubule of ER has been proposed to run through each Hechtian strand and possibly connect to the plasmodesmata [13]. These strands also contain typical cytoskeletal structures, such as microtubules, actin microfilaments, and related proteins [3,6,14]. However, it appears that these cytoskeletal structures are not required for Hechtian strand formation, as treatment with cytoskeleton inhibitors did not significantly disturb or degrade Hechtian strands [15]. Recent detailed observations revealed dynamic changes in the ER, microtubules, and Hechtian strands during plasmolysis and deplasmolysis, further indicating that these structures are tightly linked [12].

The end of a Hechtian strand, which is attached to the cell wall, forms a network-like structure known as the Hechtian reticulum [4,12]. Microscopy of plasmolyzed *Tradescantia virginiana* cells showed that the edges of the Hechtian reticulum are linked to cellulose-like fibers. In response to cellulase treatment, the Hechtian reticulum and strands disintegrate into vesicles [4], suggesting that cellulose plays critical roles in both Hechtian reticulum and strand formation. Specific proteins linking the cell wall and plasma membrane, such as glycosylphosphatidylinositol (GPI)-anchored arabinogalactan proteins [15,16], cell wall associated kinases [17], and integrin-like RGD-binding proteins [18,19], can be also involved in Hechtian strand formation. These observations suggest that Hechtian strands form physical connections between the plasma membrane and cell wall, and mechanically transduce cell wall stress signals to receptors located in the plasma membrane [8,20,21]. This Hechtian strand-mediated adhesion could mediate the transmission of piconewton-level force to the candidate molecules described above. This notion is based on the finding that the adhesion force between mammalian GPI-anchored alkaline phosphatase and the supported membrane is ~350 piconewtons [22]. However, the type of biological information that is sensed by Hechtian strands is still unknown.

In the current study, to obtain clues about the physiological roles of Hechtian strands, we employed a femtosecond (fs) laser to physically disrupt Hechtian strands in plasmolyzed plant cells. Intracellular microdissection using fs lasers is an emerging, powerful strategy used to manipulate subcellular structures and the plasma membrane without inducing photothermal damage [23,24,25]. The method has been widely used for cellular manipulation and gene delivery [23,24,25,26,27], and we previously used this method to manipulate plant cells [26,27]. Here, we specifically destroyed Hechtian strands in plasmolyzed tobacco BY-2 protoplasts by fs laser microdissection. The destruction of Hechtian strands induced the calcofluor white staining signals from β-glucans on the surfaces of protoplasts. The results of this study, representing the first study of the in vivo effects of the physical destruction of Hechtian strands, suggest the possibility that Hechtian strands transduce cell wall integrity signals between the plasma membrane and cell wall.

## 2. Results and Discussion

### 2.1. Detection of Hechtian Strands in Plasmolyzed Tobacco BY-2 Cells and Arabidopsis T87 Cells

**Figure 1:**
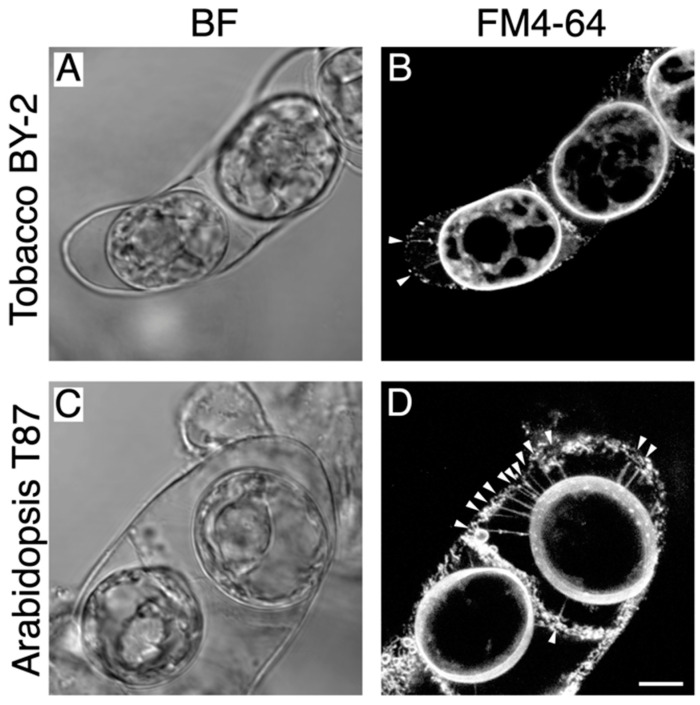
Hechtian strands in tobacco BY-2 and Arabidopsis T87 cells. Plasmolyzed tobacco BY-2 cells (**A**,**B**) and Arabidopsis T87 cells (**C**,**D**) were stained with 10 µM FM4-64 to visualize the plasma membrane and Hechtian strands (**B**,**D**). White arrowheads in (**B**,**D**) indicate Hechtian strands. BF, bright field images. Scale bar = 10 µm.

First, we established the plasmolysis conditions needed to observe Hechtian strands in tobacco (*Nicotiana tabacum*) BY-2 cells and Arabidopsis (*Arabidopsis thaliana*) T87 cells (Figure 1). After treatment with high osmotic medium containing 0.5 M mannitol, most cells underwent plasmolysis (Figure 1A,C). FM4-64 staining of these cells successfully revealed both the plasma membrane and Hechtian strands; these stretched fiber-like structures were observed in both cell types (white arrowheads in Figure 1B,D), as reported previously [12]. Only a few Hechtian strands were detected in tobacco BY-2 cells, whereas Arabidopsis T87 cells contained many Hechtian strands (Figure 1B,D). Indeed, previous studies have observed that the density of Hechtian strands differs depending on the species and cellular activity [7,8]. Therefore, the different number of Hechtian strands in tobacco BY-2 vs. Arabidopsis T87 cells possibly indicates that plasma membrane-cell wall linkages between cells could differ between species.

### 2.2. Targeted Disruption of Hechtian Strands with a Femtosecond Laser

**Figure 2:**
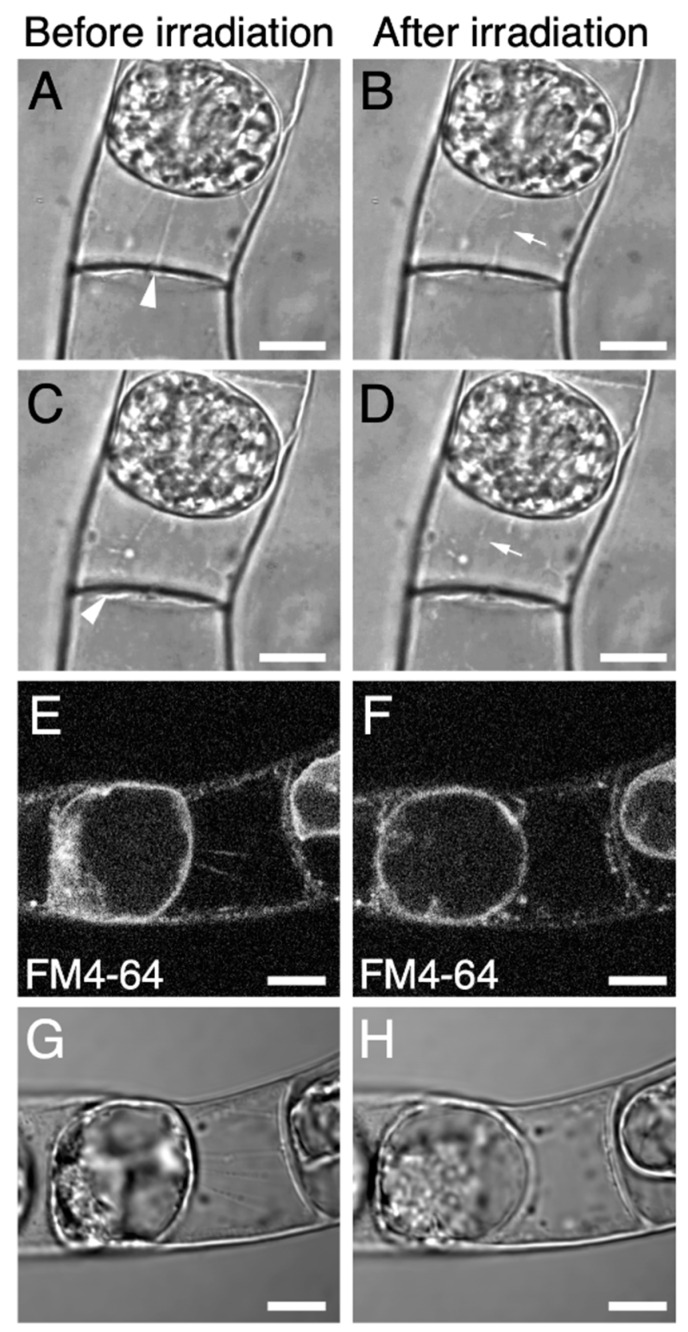
The destruction of Hechtian strands by femtosecond (fs) laser irradiation in plasmolyzed BY-2 cells. (**A**–**D**) typical images of plasmolyzed BY-2 cell before (**A**,**C**) and after (**B**,**D**) fs laser irradiation. The Hechtian strands indicated in (**A**) or (**C**) (white arrowheads) were cut in the middle of the strand by fs laser irradiation (white arrows in (**B**,**D**)). (**E**–**H**) another example of plasmolyzed BY-2 cells before (**E**,**G**) and after (**F**,**H**) fs laser irradiation. Hechtian strands were labeled with FM4-64 (**E**,**F**). Scale bars = 10 µm.

Our observations of Hechtian strands in tobacco BY-2 and Arabidopsis T87 cells (Figure 1) suggested that it would be easier to destroy Hechtian strands in BY-2 cells, as these cells have fewer Hechtian strands (Figure 1). We performed intracellular microdissection in BY-2 cells using a custom-made fs laser microdissection system (for details, please see the Section 3 and Figure S1). The fs laser pulses were focused into plasmolyzed BY-2 cells that had been stained with FM4-64 (Figure 2). We cut the middle of each Hechtian strand by fs laser irradiation (Figure 2A–D; Supplemental Movie 1) and confirmed that the signals from the Hechtian strands disappeared after irradiation (Figure 2E–H). After irradiation, no other morphological changes were detected in the cells (Figure 2B,D,F,H), indicating that our fs laser irradiation system successfully destroyed Hechtian strands without inflicting serious photothermal damage in the cells.

### 2.3. The Destruction of Hechtian Strands Enhances Cell Wall Damage Response

**Figure 3:**
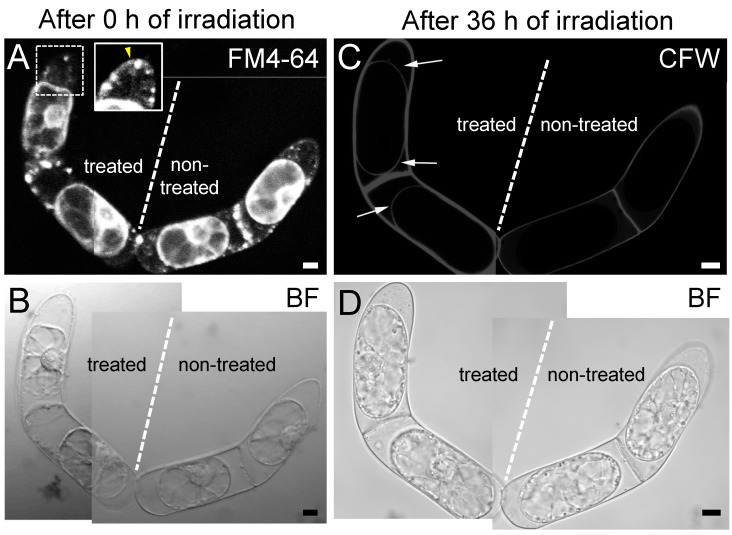
Cell wall regeneration in plasmolyzed BY-2 cells following the destruction of Hechtian strands. Of the four connected BY-2 cells examined, the two cells on the left were treated with femtosecond (fs) laser irradiation, while the two cells on the right were not treated with fs laser irradiation as a control. (**A**,**B**) the cells after 0 h of fs laser irradiation. Hechtian strands were labeled with FM4-64 (**A**). Inset in (**A**), the image before the irradiation, corresponding to the cell region marked by the square with dot line. The Hechtian strands were indicated by a yellow arrowhead. (**C**,**D**) the cells after 36 h of laser irradiation. Thin calcofluor white (CFW) signals were observed on the protoplast surface only in the two fs-irradiated cells ((**C**) white arrows). The experiment was repeated two times, and the nine fs-irradiated cells showed similar levels of enhanced cell wall regeneration. Scale bars = 10 µm.

Previous reports on the plasmolyzed *T. virginiana* leaf epidermal cells demonstrated that a fibrous meshwork containing callose and pectin was accumulated in the space between plasma membrane and the cell wall during the extended culture periods [4]. Therefore, we examined the effects of destroying Hechtian strands by fs laser irradiation on cell wall components in protoplasts (Figure 3). We selected four connected BY-2 cells (Figure 3A) and treated the two cells on the left with an fs laser to cut the Hechtian strands (Figure 3B, white arrows). The two cells on the right were not treated as a control (Figure 3B, “non-treated” cells). At 36 h after fs laser irradiation, we detected clear calcofluor white signals from β-glucans in the cell walls on the surfaces of treated protoplasts (Figure 3C, white arrows), whereas no signals were detected on the surfaces of control protoplasts (Figure 3C, “non-treated” cells). These findings strongly suggest that destroying Hechtian strands enhances the accumulation of cell wall components with β-glucans, i.e., callose or cellulose.

Hechtian strands are considered to reflect a specific type of physical adhesion between the plasma membrane and the cell wall [8,19,20]. In line with this viewpoint, the high density of Hechtian strands in tip-growing cells, such as pollen tubes and root hairs [7,8], can be explained by the notion that tip-growing cells require abundant physical connections between the plasma membrane and the cell wall to coordinate turgor pressure with apical cell wall stiffness and thus maintains proper elongation for penetration into narrow spaces [28,29]. We found that the disruption of Hechtian strands could induce the accumulation of callose and/or cellulose on the surface of protoplast in plasmolyzed cells (Figure 3). Enhancement of callose production is one of the well-known cell wall damage response in plants [30,31]. Our data cannot eliminate the possibility that the fs laser irradiation at the space between the plasma membrane and the cell wall itself would lead to cell wall damage response. However, we would like to propose that Hechtian strands can play an important role in transmitting cell wall integrity signals between the plasma membrane and cell wall (Figure 3). It would raise further possibility of fundamental changes to our current view of osmo- and mechano-sensing systems in plants, which highlights the roles of specific plasma membrane-localized channels and receptors [32,33]. Plant cells would contain specific structures that physically connect the plasma membrane and cell wall, such as Hechtian strands, to directly transmit cell wall integrity signals. Further functional analysis of Hechtian strands will provide additional insights into the mechano-sensing mechanism used by plant cells to maintain cell wall integrity for proper development and growth.

## 3. Materials and Methods

### 3.1. Plant Cell Materials and Growth Conditions

Suspension cultures of tobacco (*Nicotiana tabacum*) BY-2 and Arabidopsis (*Arabidopsis thaliana*) T87 cells were maintained in modified Linsmaier and Skoog medium containing Murashige and Skoog plant salt mixture (Wako Pure Chemical Industries Ltd., Osaka, Japan) [34,35]. The tobacco BY-2 cells were maintained in a rotary shaker at 130 rpm at 27 °C in the dark, and the Arabidopsis T87 cells were maintained in a 130 rpm rotary shaker at 22 °C in the light. Every week, the tobacco BY-2 cells were diluted 100-fold and the Arabidopsis T87 cells were diluted 20-fold in fresh liquid medium [34,35].

### 3.2. Plasmolysis Treatment

For plasmolysis, the tobacco BY-2 and Arabidopsis T87 suspension cells were collected by gentle centrifugation (100 rpm, 1 min, at room temperature) and resuspended in modified Linsmaier and Skoog medium containing 0.5 M mannitol. The cells with or without femtosecond laser treatment were incubated in a growth chamber without shaking at 27 °C in the dark (tobacco) and 22 °C in the light (Arabidopsis).

### 3.3. Fluorescent Dye Labeling of Cells

To label plasma membrane and Hechtian strands, 10 µM FM4-64 (ThermoFisher Scientific, MA, USA) was added to the cell culture medium. After 10 min of incubation, we observed the cells. To stain the cell wall (cellulose and/or callose), calcofluor white was added to the cell culture medium at a final concentration of 10 mg/L. The stained cells were transferred onto φ35-mm Petri dishes with a φ14-mm coverslip window at the bottom (Matsunami Glass Industries, Ltd., Osaka, Japan).

### 3.4. Confocal Laser-Scanning Microscopy and Image Processing

The stained cells were observed under an inverted fluorescence microscope (AxioVert 200M; Zeiss, Oberkochen, Germany) equipped with a confocal laser scanning head system (LSM 710; Zeiss), or the inverted platform of a fluorescence microscope (BX53; Olympus Co. Ltd., Tokyo, Japan) equipped with an FV1000 confocal scanning system (Olympus, Tokyo, Japan). The images were digitally processed with ImageJ (http://rsb.info.nih.gov/ij/) [36] or Adobe Photoshop (version CS4; Adobe Systems Inc., Mountain View, CA, USA).

### 3.5. Intracellular Microdissection with an fs Laser Amplifier

Femtosecond laser pulses (800 nm, 150 fs) from a regeneratively amplified femtosecond Ti:Sapphire laser amplifier (Solstice; Spectra Physics Co. Ltd., Santa Clara, CA, USA) were delivered to samples under an inverted microscope (IX71; Olympus, Tokyo, Japan) equipped with a confocal laser-scanning system (FV300; Olympus, Tokyo, Japan). After placing the sample on the motorized stage of the microscope, a single laser pulse was extracted with a mechanical shutter with a gate time of 1/32 s from 32 Hz pulse trains and focused on the cell through a 100× oil-immersion objective (PlanN NA:1.25; Olympus, Tokyo, Japan). The laser pulse energy was tuned to be 20 nJ/pulse.

For the femtosecond (fs) laser treatment, we used a glass bottom dish with a Poly-L-lysine coating, where a silicon film with a 5 mm-square hole was placed. The glass bottom dish was marked by a small cross mark with the fs laser, and this cross mark was used for the recognition of cell positions. The cell culture solution with 10~20 plasmolyzed tobacco BY-2 cells was placed in the hole of silicon film and covered with a cover glass. After the disruption of Hechtian strands for selected 3–4 cells, the cells were cultured in a growth chamber without shaking at 27 °C in the dark carefully. We repeated the fs irradiation experiments more than 20 times.

## Supplementary Materials

The following materials are available online at https://www.mdpi.com/2223-7747/9/5/604/s1, Figure S1: Femtosecond laser microdissection system, Supplemental Movie 1: Destruction of Hechtian strands by femtosecond laser irradiation.
