# Supplementary material for: Hechtian Strands Transmit Cell Wall Integrity Signals in Plant Cells
Source: Plants (Basel). 2020 May 9;9(5):604. doi: 10.3390/plants9050604 (PMC7284614; doi:10.3390/plants9050604)
Supplement: Supplementary file 1 [file plants-09-00604-s001.zip › Yoneda et al Suppl/SupFigS1.pdf]

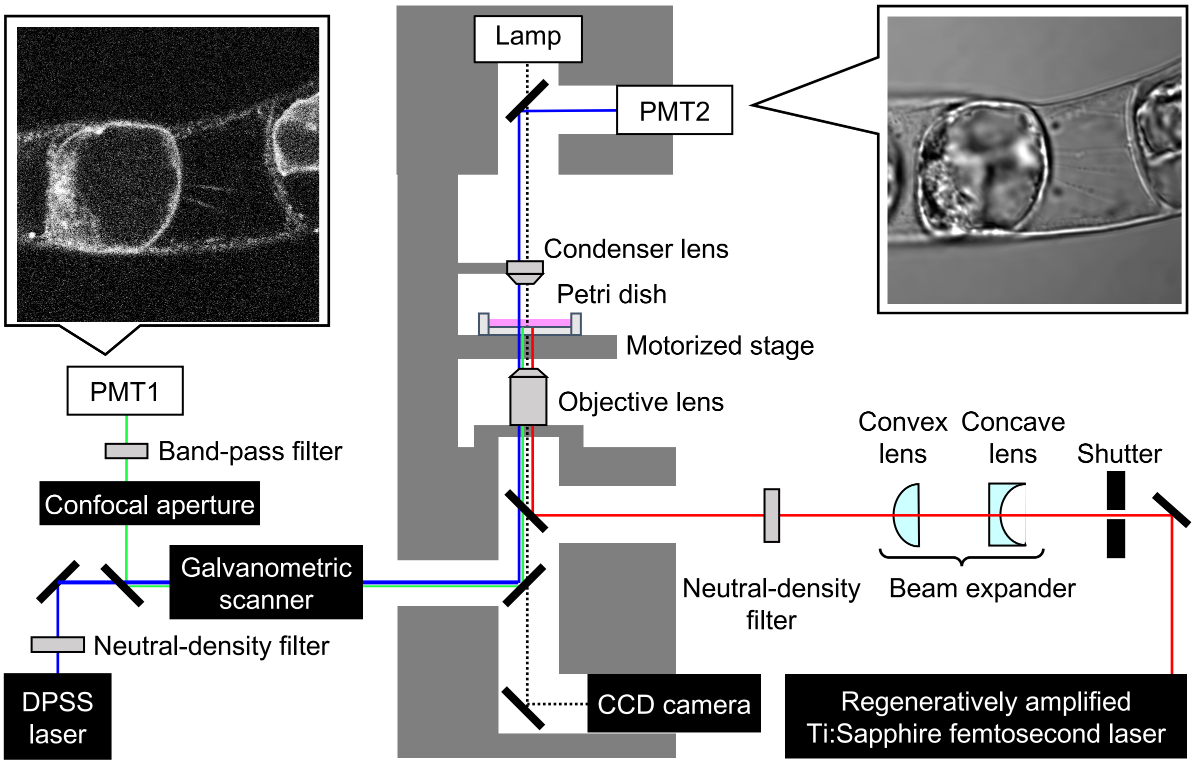

Supplemental Figure 1. Femtosecond laser microdissection system.
Femtosecond laser light (800 nm, 150 fs) was introduced into a confocal laser-scanning microscope and focused on a tobacco BY-2 cell through an objective lens (20×, N.A. 0.46). Cells in the Petri dish were illuminated by a lamp producing white light and monitored using a CCD camera. Confocal laser-scanning microscopy of the cells was performed using a DPSS laser, a laser scanner, a band-pass filter, and a photomultiplier tube (PMT).
